# Understanding Patterns of Adherence to Antifibrotic Treatment in Idiopathic Pulmonary Fibrosis: Insights from an Italian Prospective Cohort Study

**DOI:** 10.3390/jcm13092727

**Published:** 2024-05-06

**Authors:** Marica Iommi, Francesca Gonnelli, Martina Bonifazi, Andrea Faragalli, Federico Mei, Marco Pompili, Flavia Carle, Rosaria Gesuita

**Affiliations:** 1 Center of Epidemiology, Biostatistics and Medical Information Technology, Università Politecnica delle Marche, 60126 Ancona, Italy; m.iommi@staff.univpm.it (M.I.); f.carle@staff.univpm.it (F.C.); r.gesuita@staff.univpm.it (R.G.); 2 Department of Biomedical Sciences and Public Health, Università Politecnica delle Marche, 60126 Ancona, Italy; f.gonnelli@pm.univpm.it (F.G.); m.bonifazi@staff.univpm.it (M.B.); f.mei@staff.univpm.it (F.M.); 3 Interstitial Lung Diseases, Pleural Diseases and Bronchiectasis Unit, Azienda Ospedaliero-Universitaria “Ospedali Riuniti”, 60126 Ancona, Italy; 4 Regional Health Agency of Marche, 60121 Ancona, Italy; marco.pompili@regione.marche.it; 5 National Centre for Healthcare Research and Pharmacoepidemiology, 20126 Milan, Italy; 6 Istituto di Ricovero e Cura a Carattere Scientifico Istituto Nazionale di Ricovero e Cura per Anziani, 60121 Ancona, Italy

**Keywords:** idiopathic pulmonary fibrosis, antifibrotic treatment, drug utilization pattern, adherence, Healthcare Utilization Databases

## Abstract

**Background**: Pirfenidone and Nintedanib have significantly improved the prognosis of patients with idiopathic pulmonary fibrosis (IPF), reducing mortality risk and exacerbations. This study aimed to analyze antifibrotic treatment utilization and its association with clinical outcomes (i.e., acute exacerbation or death) during 2014–2021 in newly diagnosed IPF patients, using Healthcare Utilization Databases of the Marche Region, Italy. **Methods**: The first 12-month adherence to antifibrotic was estimated using the Proportion of Days Covered (PDC), defining adherence as PDC ≥ 75%. State Sequence Analysis over the initial 52 weeks of treatment was used to identify adherence patterns. The role of adherence patterns on acute exacerbations/death, adjusted by demographic, clinical features, and monthly adherence after the 52-week period (time-dependent variable), was assessed with Cox regression. **Results**: Among 667 new IPF cases, 296 received antifibrotic prescriptions, with 62.8% being adherent in the first year. Three antifibrotic utilization patterns emerged—high adherence (37.2%), medium adherence (42.5%), and low adherence (20.3%)—with median PDCs of 95.3%, 79.5%, and 18.6%, respectively. These patterns did not directly influence three-year mortality/exacerbation probability, but sustained adherence reduced risk over time. **Conclusions**: Good adherence was observed in in this population-based study, emphasizing the importance of continuous antifibrotics therapy over time to mitigate adverse outcomes.

## 1. Introduction

Idiopathic pulmonary fibrosis (IPF) is a rare, chronic, and progressive disease that causes fibrosis in the lungs. IPF predominantly occurs in elderly individuals, and it manifests through a progression of breathlessness and declining lung function, leading to an unfavorable prognosis [1]. In Italy, two drugs are currently available, Pirfenidone and Nintedanib, approved by the Italian Medicine Agency and fully entered in clinical practice in 2014 and 2016, respectively. These drugs changed the therapeutic management of the disease by improving patient prognosis and reducing the risk of mortality and acute exacerbations by 45% and 37%, respectively [2].

In clinical practice, Pirfenidone can be prescribed to patients under 81 years of age with early or moderate disease severity, Forced Vital Capacity (FVC) ≥50%, and diffusing capacity of the lung for carbon monoxide (DLCO) ≥ 35%. Nintedanib is indicated for the treatment of IPF within similar functional thresholds (FVC ≥ 50%, DLCO ≥ 30%), but it is also allowed over 81 years of age.

Clinical trials have demonstrated the efficacy of both antifibrotics in reducing the progression of the disease [3,4], although reporting a high frequency of common adverse events (diarrhoea, nausea, vomiting, weight loss), which might lead to a dose reduction, a shift towards the other drug, or treatment discontinuation. Although both drugs have shown comparable efficacy [5] and no clear impact of dose reduction on survival has been demonstrated [6], drug withdrawal was associated with a statistically significant more rapid functional decline [7].

Furthermore, adherence to therapy is a required condition for the antifibrotics to be effective, but only a few studies have evaluated the adherence pattern in real life so far [8,9,10].

Secondary data sources, in particular Healthcare Utilization Databases (HUD), allow for rapidly describing the pattern of drug utilization over time in large, unselected populations [11].

Therefore, the aim of this study was to investigate the utilization patterns of antifibrotic treatment and their association with clinical outcomes (i.e., acute exacerbation or death) during 2014–2021 in patients with newly diagnosed IPF between 2014 and 2019 using Healthcare Utilization Databases.

## 2. Materials and Methods

### 2.1. Study Population, Data Sources, and Incident IPF Case Definition

In this prospective, population-based study, data were obtained from Healthcare Utilization Databases of Marche, a region in central Italy with a target population of about 1.3 million inhabitants, during a study period between 2014 and 2021.

The secondary data sources used were the Regional Beneficiaries database (RBD), Hospital Discharge Records (HDR), the Drug prescriptions (DP) database, and the Outpatient care database (OCD). The algorithm for identifying new cases of IPF has been previously described [12]. We included all of the subjects residing in the Marche Region at their first hospital discharge with an ICD-9-CM code of 516.3 in the primary or secondary diagnosis fields or at their first prescription of Pirfenidone or Nintedanib, between 1 January 2014 and 31 December 2019 (index date). For the purposes of the analysis, subjects with a follow-up period shorter than 30 days, residing in the Marche Region for less than 3 years before the index date, or with a hospitalization for IPF or an antifibrotic prescription in the period of 2011–2013, were excluded. Patients diagnosed with lung cancer within three years prior to the index date were excluded.

### 2.2. Antifibrotics Utilization Pattern

The adherence to an antifibrotic prescription was estimated using the Proportion of Days Covered (PDC) [13], i.e., the proportion of days in which a person has access to the antifibrotic drug over a period of 12 months after the first prescription. Patients were considered adherent if PDC ≥ 75%.

In addition, the weekly PDC metric was calculated by dividing the 12-month period after the first prescription into 52 weeks to assess the individual’s annual pattern of antifibrotic adherence. For the purpose of this analysis, we included in this analysis only patients who were alive during the first 12 months after the first prescription.

Patients’ antifibrotic intolerance was defined on the basis of two consecutive prescriptions reporting a switch from Pirfenidone to Nintedanib or from Nintedanib to Pirfenidone or a dose reduction from 150 mg to 100 mg of Nintedanib within 12 months from the first prescription. For the antifibrotic intolerance analysis, only subjects with a first antifibrotic prescription since 1 January 2016 were considered, as Nintedanib was not available before.

### 2.3. Statistical Analysis

For descriptive purposes, the cumulative probability of receiving the first antifibrotic prescription from the index date was evaluated considering new cases of IPF identified from the HUD in the study period and applying the Kaplan Meier method.

The proportion of patients with PDC ≥ 75% was estimated by calculating the 95% confidence interval (95% CI) and stratifying by sex, age (dichotomized at 75 years), and Multisource Comorbidity Score (MCS), a method based on HDR and DP databases for assessing subjects’ health conditions in the two years preceding the index date [14]. The MCS was categorized into two classes of 0–4, good or fair health condition, and ≥5, slightly poor or poor health condition.

A logistic regression model was used to estimate the association of antifibrotic prescription adherence with sex, age, MCS, and the number of concomitant drugs in addition to the antifibrotics, based on the distinct 7th ATC code level, during the first year from the first antifibrotic prescription. The results were expressed as Odds Ratio (OR) along with 95% CI.

The State Sequence Analysis [15] (SSA) was used to describe the pattern of use of antifibrotics by an individual through weekly adherence sequences. States were defined by the weekly PDC ≥ 75% (yes or not) observed over 52 weeks from the first prescription; the succession of states over time defined the entire antifibrotic utilization pattern of each subject (individual sequence). The analysis consisted of the two-by-two comparison of sequences, the definition of a similarity (or dissimilarity) measure, and the clustering of the sequences. The dissimilarity matrix, containing the distances between each pair of sequences from two different subjects, was obtained using the Longest Common Subsequence (LCS) metric, in which two different sequences are considered similar based on the length of the parts (subsequences) shared. The dissimilarity matrix was then used for identifying common adherence patterns by means of cluster analysis. The demographic, clinical, and adherence characteristics were compared between patterns of use of antifibrotics defined in the State Sequence Analysis using the chi-squared test and the Kruskal–Wallis test, as appropriate.

We included in the SSA only the sub-cohort of individuals who were alive and without acute exacerbations at 52 weeks after the first prescription. The 52-week period was chosen because a high survival or acute exacerbation-free probability in patients receiving at least one antifibrotic prescription was previously reported [16]. Acute exacerbations were defined as all respiratory-related events requiring hospitalization, as previously described [16].

Multiple Cox regression analysis was performed to evaluate the role of adherence patterns on the composite outcome, acute exacerbations/death, adjusted by sex, age, and MCS assessed at the end of the 52-week period, and monthly adherence status (PDC ≥ 75%) evaluated after the first year from the first drug prescription, included in the model as a time-dependent variable.

The probability of antifibrotic intolerance at 12 months from the first prescription was estimating considering new IPF cases detected between 2016 and 2019 and using the Kaplan–Meier curve and multiple Cox regression model, adjusted by sex, age, and MCS. The results were expressed as hazard ratios (HR) and 95% CI.

The significance level for all of the analyses was set at *p* < 0.05. Statistical analyses were performed using R, version 4.2.1.

## 3. Results

The cohort of new IPF diagnosis included 667 patients from 2014 to 2019. Among them, 489 (73.3%) were identified from the HDR. In this sub-cohort, 126 subjects received at least one prescription of antifibrotic, and the probability of entering treatment at five years of follow-up was 30.1% (95% CI: 24.7–35.0), with a median time of 59 days (interquartile range: 36.3–184.3).

### 3.1. Adherence to IPF therapy

A total of 296 (44.4%) patients with IPF received at least a prescription of an antifibrotic during 2014–2019. In total, 278 (77.0%) patients were males, 123 (41.6%) had an age higher than or equal to 75 years, and 121 (40.9%) had slightly poor or poor health conditions at the index date.

Overall, 186 patients (62.8%) were adherent to antifibrotic therapy at the end of the first year of therapy. The probability of adherence was not significantly associated with baseline demographic and clinical features, nor with the number of concomitant drugs (Table 1). No difference between Pirfenidone and Nintedanib was observed in the probability of adherence (OR: 1.06; 95% CI: 0.93–1.21) in the period of 2016–2019 when both of the two drugs were available in Italy.

### 3.2. State Sequence Analysis

Three patterns of antifibrotic utilization were identified through the SSA performed on 261 of 296 patients with at least one drug prescription; in total, 35 subjects were excluded from the analysis because they died or experienced acute exacerbations within the 52-week period.

**Figure 1:**
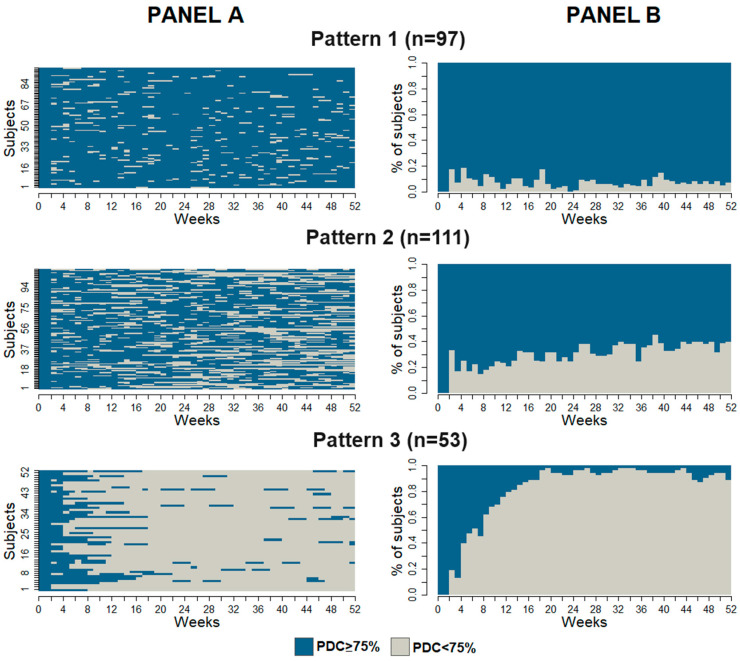
Index plots for weekly adherence (**Panel A**) and evolution of the proportion of patients adhering to antifibrotic therapy (**Panel B**) in the 52 weeks after the first drug prescription.

In Figure 1 Panel A, the individual sequences are reported for each pattern identified, in which the 52 weeks after the first drug prescription are on the abscissa and the subjects on the ordinate. Blue subsequences of each subject’s sequence indicate the states in which each subject was adherent, while grey subsequences indicate the weeks with a PDC < 75%. Pattern 2 was predominant (n = 111, 42.5%) and included subjects with a medium level of adherence; Patterns 1 and 3 included 97 (37.2%) and 53 (20.3%) subjects, respectively, with the highest and lowest levels of adherence. Figure 1 Panel B shows the proportion of adherent patients (blue bars) in each week for the three patterns. In Pattern 1, this proportion can be considered stable and high, while in the other two patterns, it decreases over the 52 weeks, and more rapidly in Pattern 3.

The main characteristics of subjects according to the three patterns are reported in Table 2. Their clinical and demographic features were comparable. As expected, no subject with PDC ≥ 75% in the 12 months following the first prescription was found in the Pattern 3 group, while 55.9% of subjects were adherent in Pattern 2. The distribution of PDC values was significantly higher in Pattern 1 than in the other two clusters. In particular, in Pattern 3, 75% of subjects had a PDC value equal to or below 29.9%.

Table 3 shows the results of the Cox regression analysis. The pattern of adherence to antifibrotic treatment within 52 weeks of the first prescription did not significantly affect the probability of dying or developing acute exacerbations at three years after the initial drug prescription (number of events: 57); nevertheless, the longer subjects adhered to treatment, the lower the risk of dying or developing acute exacerbations, by about 60% (HR = 0.39, 95% CI: 0.18–0.86). Also, subjects in slightly poor or poor health conditions had a risk of developing the composite outcome 1.92 times higher than those in good or fair health conditions (95% CI: 1.05–3.53).

### 3.3. Intolerance to Antifibrotic Therapy

A total of 258 patients started antifibrotic treatment since 2016 and, among them, 65 patients developed intolerance to antifibrotic therapy at 12 months, with a cumulative probability of 25.4% (95% CI: 19.9–30.6).

Of the 143 (55.4%) patients who had Pirfenidone as first-line therapy, 19 switched to Nintedanib over a 12-month period, with a probability of intolerance of 13.4% (95% CI: 7.6–18.8), and only 1 patient switched from Nintedanib to Pirfenidone amongst patients aged <81 years (n = 93). Twenty-two patients started the treatment with Nintedanib, and they could not switch to Pirfenidone because they were 81 years old or older.

A dose reduction from 150 mg to 100 mg of Nintedanib within 12 months from the first prescription was observed in 45 out of 107 patients who started with Nintedanib (42.4%, 95% CI: 32.2–51.1).

The probability of developing drug intolerance was significantly higher in females, in older subjects, and in those with a slightly poor/poor health condition (Table 4).

## 4. Discussion

The present population-based study firstly provided real-world evidence of adherence to antifibrotics within the first year of treatment in patients with IPF using secondary data sources, such as Healthcare Utilization Databases. About 63% of patients had a proportion of days covered ≥75% in the first year of treatment, without differences in sex, age, or health conditions at IPF diagnosis. Furthermore, the level of adherence was similar between patients treated with Pirfenidone and those treated with Nintedanib, but the risk of drug intolerance was higher among females, the elderly, and patients in slightly poor/poor conditions. These results are consistent with those reported from previous real-world observational studies using primary data sources, confirming the reliability of our methodological approach [8,17].

Three distinct utilization patterns emerged from the State Sequence Analysis, capturing the adherence level over time in the first 52 weeks of treatment. Over 37% of patients presented an optimal level of adherence (Pattern 1) and 43% a satisfactory one (Pattern 2). In total, 20.3% of subjects had poor adherence, and most of them discontinued the treatment within the first few weeks from its initiation (Pattern 3). Similar results were also detected by Nili et al. [18], who analyzed adherence to Nintedanib among patients with IPF and reported that early poor adherence to Nintedanib occurred in 21.5% of the analyzed subjects. Consistently with Nili et al. [18], we also observed that the frequency of subjects aged ≥75 and in slightly poor/poor health condition was higher in the Pattern 3 group than the other two groups, at a level of probability of 7%. A possible explanation is that the severity of the disease, the frequent occurrence of side effects, as well as socio-economic status and level of education may play a role on the probability of having an early poor pattern of antifibrotic utilization.

We did not detect any difference between the adherence pattern and the type of antifibrotic drug. This is apparently in contrast to what Wright et al. found in their retrospective analysis [19]. However, they observed a lower tolerance to Pirfenidone than Nintedanib only in the very first month of follow-up, with a parallel adherence pattern thereafter, which is consistent with our results.

We provided real-world evidence about the diffusion of the antifibrotic drugs, finding that 44% of patients with newly diagnosed IPF were prescribed these drugs during the study period. This is a relatively high number of patients, considering that the very first period since the Italian Drug Agency approval of these drugs was included in the analysis. In fact, Moor et al. [20] assessed the widespread diffusion of the antifibrotic therapy across Europe using a survey to health care practitioners and patients and found a considerably high percentage of treated patients (82%), but their study was published in 2019, which is at the end of our study period, and the diffusion of the drug is supposed to increase over time.

Several studies aiming at confirming the protective role of the antifibrotic drugs have recently been published. Behr et al. [21] prospectively analyzed IPF patients, assessing the 2-year risk of mortality in patients treated with antifibrotic vs. non-treated patients, and they found a 37% lower risk of death among treated patients independent of lung function decline. Likewise, in a metanalysis by Petnak et al. [2], they detected inferior acute exacerbation and death rates among patients with IPF who were under antifibrotic treatments, and the evidence was stronger for Nintedanib than Pirfenidone. These data confirmed the protective role of these drugs but did not provide any evidence regarding the importance of treatment adherence in the real world and how it might affect the effectiveness of these drugs in daily clinical practice.

### Strengths and Weaknesses

This study has some limitations. First, the evaluation of adherence based on Healthcare Utilization Databases has the downside that prescription refilling patterns might differ from the actual patient’s medication-taking behavior [22]. However, this method is rapid and easy to use, inexpensive, does not directly involve patients, produces evidence on adherence from HUD-based studies, and can provide important methodological suggestions to healthcare managers for continuous monitoring and evaluation of treatment adherence. Second, HUD do not collect some important clinical predictors of antifibrotic adherence, such as the occurrence of side effects, the severity of the disease, and other patient clinical characteristics that condition patients’ behavior towards the treatment and contribute to explaining the different utilization profiles identified in this study. Third, imprecision regarding the exact timing of the IPF diagnosis must be acknowledged: new cases are detected either during their first IPF-related hospitalization or upon receiving their first prescription of antifibrotics, and these events might happen several months after the outpatient diagnostic visit. Furthermore, in using healthcare administrative databases, a misclassification of the diagnosis might occur; however, we focused on antifibrotic users, and until 2021, antifibrotic drugs were only indicated by the Italian Medicine Agency for patients with IPF or with lung cancer, and the latter were excluded from the analyses.

Among the study’s strengths, the results were obtained in a real-world setting of an unselected population. The use of HUD allowed us to describe and analyze the adherence and tolerance to an antifibrotic prescription in patients with IPF, and both are in line with real-world data derived from cohort hospital-based studies. Moreover, the State Sequence Analysis applied to analyze the use of antifibrotics over time identified three main patterns of drug use, which are easy to read and interpret and based on individual behavior towards therapy.

## 5. Conclusions

In this context, our findings indicate that two-thirds of newly diagnosed IPF patients were adherent to an antifibrotic during the initial year of treatment. Moreover, maintaining a proportion of time covered by the antifibrotic of at least 75% over time decreases the probability of death or acute exacerbations, whereas we did not find this protective effect at lower levels of adherence. These results are of paramount importance, especially given that over a third of treated patients will not achieve the expected reduction of death and acute exacerbation risk. In addition, our study highlights the need for enhanced supervision and monitoring of patients classified as poorly adherent (Pattern 3). Further research would be valuable for healthcare professionals managing these patients in order to detect determinants of non-adherence at an early stage and to provide tailored interventions to improve adherence.

## Figures and Tables

**Table 1:** Number and percentage of adherent patients and association between adherence and sex, age, MCS, and number of concomitant drugs (n = 296). Results of multiple logistic regression model.

	Adherence		OR (95%CI)
	n	% (95% CI)	
PDC ≥ 75%	186	62.8 (57.0–68.3)	
**Sex**			
Female	42	61.8 (49.1–73.0)	1
Male	144	63.2 (56.5–69.4)	1.02 (0.58–1.79)
**Age**			
<75 years	112	64.7 (57.1–71.7)	1
≥75 years	74	60.2 (50.9–68.8)	0.88 (0.54–1.44)
**MCS at baseline**			
Good/fair health condition	114	65.1 (57.5–72.0)	1
Slightly poor/poor health condition	72	59.5 (50.2–68.2)	0.86 (0.52–1.42)
**Number of concomitant** **drugs, median (IQR) ^1^**	186	8 (5–12)	0.97 (0.92–1.02)

PDC: Proportion of Days Covered; MCS: Multisource Comorbidity Score; IQR: interquartile range; OR: Odds Ratio; 95% CI: 95% confidence interval. ^1^ Number of concomitant drugs in addition to the antifibrotics, based on the distinct 7th ATC code level, during the first year from the first antifibrotic prescription; in non-adherent patients, the median number of concomitant drugs was 9 (IQR 6–13).

**Table 2:** Adherence and demographic and clinical characteristics of the subjects by pattern of use of antifibrotics defined in the State Sequence Analysis.

n (%)	Total	Pattern 1	Pattern 2	Pattern 3	*p*
	(n = 261)	(n = 97)	(n = 111)	(n = 53)	
PDC ≥ 75% ^1^	159 (60.9%)	97 (100%)	62 (55.9%)	0 (0%)	<0.001 ^a^
PDC ^2^, median (IQR)	84.7 (56.4–93.4)	95.3 (93.2–97.5)	79.5 (65.9–86.2)	18.6 (9.3–29.9)	<0.001 ^b^
Females	61 (23.4%)	23 (23.7%)	26 (23.4%)	12 (22.6%)	0.989 ^a^
Age ≥ 75 years	112 (42.9%)	40 (41.2%)	42 (37.8%)	30 (56.6%)	0.069 ^a^
Slightly poor/poor health condition ^3^	160 (61.3%)	54 (55.7%)	67 (60.4%)	39 (73.6%)	0.095 ^a^
Number of concomitant drugs, median (IQR) ^4^	8 (5–12)	8 (5–11)	8 (5–12)	9 (6–13)	0.421 ^b^
Nintedanib ^5^	99 (37.9%)	33 (34.0%)	44 (39.6%)	22 (41.5%)	0.590 ^a^
Acute exacerbations	64 (24.5%)	21 (21.6%)	28 (25.2%)	15 (28.3%)	0.647 ^a^

^1^ Number of subjects with a PDC ≥ 75% over the first 12 months after the first prescription. ^2^ PDC median value over the first 12 months after the first prescription. ^3^ Assessed at the end of the 52-week period. Slightly poor/poor health condition: Multisource Comorbidity Score ≥5. ^4^ Number of concomitant drugs in addition to the antifibrotics, based on distinct 7th ATC code level, during the first year from the first antifibrotic prescription. ^5^ Antifibrotic received in the first prescription. PDC: Proportion of Days Covered. ^a^ Chi-squared test. ^b^ Kruskal–Wallis test.

**Table 3:** Probability of dying or developing acute exacerbations at three years from first drug prescription. Cox regression analysis results.

	HR	95 % CI		*p*
Age (≥75 vs. <75 years)	1.53	0.91	2.59	0.109
Sex (males vs. females)	1.16	0.61	2.20	0.646
Pattern of adherence (medium vs. high)	0.74	0.34	1.60	0.438
Pattern of adherence (low vs. high)	0.55	0.20	1.52	0.250
Health conditions (slightly poor/poor vs. good/fair) ^1^	1.92	1.05	3.53	0.035
Monthly adherence (PDC ≥ 75% vs. <75%) ^2^	0.39	0.18	0.86	0.019

^1^ Assessed at the end of a 52-week period with MCS, Multisource Comorbidity Score. ^2^ Monthly adherence status (Proportion of Days Covered (PDC) ≥ 75%) evaluated after the first year from the first drug prescription, included in the model as time-dependent. HR: Hazard Ratio; 95% CI: 95% confidence interval.

**Table 4:** Factors associated with antifibrotic intolerance. Results of Cox regression analysis.

	HR	95% CI		*p*
Age (≥75 vs. <75 years)	1.64	1.01	2.65	0.045
Sex (males vs. females)	0.45	0.27	0.75	0.002
Health conditions (slightly poor/poor vs. good/fair) ^1^	2.11	1.28	3.46	0.003

^1^ Assessed with MCS, Multisource Comorbidity Score. HR: Hazard Ratio; 95% CI: 95% confidence interval.

## Data Availability

Restrictions apply to the availability of these data. Data were obtained from the Marche Region and are available with the permission of the Marche Region.
